# Immunogenetics of lithium response and psychiatric phenotypes in patients with bipolar disorder

**DOI:** 10.1038/s41398-024-02865-4

**Published:** 2024-04-03

**Authors:** Marisol Herrera-Rivero, Karina Gutiérrez-Fragoso, International Consortium on Lithium Genetics (ConLi+Gen), Anbupalam Thalamuthu, Azmeraw T. Amare, Mazda Adli, Kazufumi Akiyama, Nirmala Akula, Raffaella Ardau, Bárbara Arias, Jean-Michel Aubry, Lena Backlund, Frank Bellivier, Antonio Benabarre, Susanne Bengesser, Abesh Kumar Bhattacharjee, Joanna M. Biernacka, Armin Birner, Micah Cearns, Pablo Cervantes, Hsi-Chung Chen, Caterina Chillotti, Sven Cichon, Scott R. Clark, Francesc Colom, Cristiana Cruceanu, Piotr M. Czerski, Nina Dalkner, Franziska Degenhardt, Maria Del Zompo, J. Raymond DePaulo, Bruno Etain, Peter Falkai, Ewa Ferensztajn-Rochowiak, Andreas J. Forstner, Josef Frank, Louise Frisén, Mark A. Frye, Janice M. Fullerton, Carla Gallo, Sébastien Gard, Julie S. Garnham, Fernando S. Goes, Maria Grigoroiu-Serbanescu, Paul Grof, Ryota Hashimoto, Roland Hasler, Joanna Hauser, Urs Heilbronner, Stefan Herms, Per Hoffmann, Liping Hou, Yi-Hsiang Hsu, Stéphane Jamain, Esther Jiménez, Jean-Pierre Kahn, Layla Kassem, Tadafumi Kato, John Kelsoe, Sarah Kittel-Schneider, Po-Hsiu Kuo, Ichiro Kusumi, Barbara König, Gonzalo Laje, Mikael Landén, Catharina Lavebratt, Marion Leboyer, Susan G. Leckband, Mario Maj, Mirko Manchia, Cynthia Marie-Claire, Lina Martinsson, Michael J. McCarthy, Susan L. McElroy, Vincent Millischer, Marina Mitjans, Francis M. Mondimore, Palmiero Monteleone, Caroline M. Nievergelt, Tomas Novák, Markus M. Nöthen, Claire O’Donovan, Norio Ozaki, Sergi Papiol, Andrea Pfennig, Claudia Pisanu, James B. Potash, Andreas Reif, Eva Reininghaus, Hélène Richard-Lepouriel, Gloria Roberts, Guy A. Rouleau, Janusz K. Rybakowski, Martin Schalling, Peter R. Schofield, Klaus Oliver Schubert, Eva C. Schulte, Barbara W. Schweizer, Giovanni Severino, Tatyana Shekhtman, Paul D. Shilling, Katzutaka Shimoda, Christian Simhandl, Claire M. Slaney, Alessio Squassina, Thomas Stamm, Pavla Stopkova, Fabian Streit, Fasil Tekola-Ayele, Alfonso Tortorella, Gustavo Turecki, Julia Veeh, Eduard Vieta, Biju Viswanath, Stephanie H. Witt, Peter P. Zandi, Martin Alda, Michael Bauer, Francis J. McMahon, Philip B. Mitchell, Marcella Rietschel, Thomas G. Schulze, Joachim Kurtz, Bernhard T. Baune

**Affiliations:** 1 Department of Psychiatry and Psychotherapy, University of Münster, Münster, Germany; 2 Department of Genetic Epidemiology, Institute of Human Genetics, University of Münster, Münster, Germany; 3 Joint Institute for Individualisation in a Changing Environment (JICE), University of Münster and Bielefeld University, Münster, Germany; 4 Division of Engineering in Computational Systems, Higher Technological Institute of the East of the State of Hidalgo, Hidalgo, Mexico; 5 Institute for Evolution and Biodiversity, University of Münster, Münster, Germany; 6 Department of Psychiatry, Melbourne Medical School, University of Melbourne, Melbourne, Australia; 7 The Florey Institute of Neuroscience and Mental Health, The University of Melbourne, Melbourne, Australia; 8 Centre for Healthy Brain Ageing (CHeBA), School of Psychiatry, University of New South Wales, Sydney, Australia; 9 Discipline of Psychiatry, School of Medicine, University of Adelaide, Adelaide, SA Australia; 10 Department of Psychiatry and Psychotherapy, Charité - Universitätsmedizin Berlin, Campus Charité Mitte, Berlin, Germany; 11 Fliedner Klinik Berlin, Berlin, Germany; 12 Department of Biological Psychiatry and Neuroscience, Dokkyo Medical University School of Medicine, Tochigi, Japan; 13 Intramural Research Program, National Institute of Mental Health, National Institutes of Health, US Department of Health & Human Services, Bethesda, USA; 14 Unit of Clinical Pharmacology, Hospital University Agency of Cagliari, Selargius, Italy; 15 Unitat de Zoologia i Antropologia Biològica (Dpt. Biologia Evolutiva, Ecologia i Ciències Ambientals), Facultat de Biologia, University of Barcelona, CIBERSAM, Barcelona, Spain; 16 Institut de Biomedicina (IBUB), University of Barcelona, CIBERSAM, Barcelona, Spain; 17 Department of Psychiatry, Division of Psychiatric Specialities, Geneva University Hospitals, Geneva, Switzerland; 18 Faculty of Medicine, University of Geneva, Geneva, Switzerland; 19 Department of Molecular Medicine and Surgery, Karolinska Institute, Solna, Sweden; 20 Center for Molecular Medicine, Karolinska University Hospital, Stockholm, Sweden; 21 INSERM UMR-S 1144, Université Paris Cité, Paris, France; 22 Département de Psychiatrie et de Médecine Addictologique, AP-HP, Groupe Hospitalier Saint-Louis-Lariboisière-F. Widal, Paris, France; 23 Bipolar Disorder Program, Institute of Neuroscience, Hospital Clinic, University of Barcelona, IDIBAPS, CIBERSAM, Barcelona, Spain; 24 Department of Psychiatry and Psychotherapeutic Medicine, Research Unit for Bipolar Affective Disorder, Medical University of Graz, Graz, Austria; 25 Department of Psychiatry, University of California San Diego, San Diego, USA; 26 Department of Health Sciences Research, Mayo Clinic, Rochester, USA; 27 Department of Psychiatry and Psychology, Mayo Clinic, Rochester, USA; 28 The Neuromodulation Unit, McGill University Health Centre, Montreal, Canada; 29 Department of Psychiatry & Center of Sleep Disorders, National Taiwan University Hospital, Taipei City, Taiwan; 30 Human Genomics Research Group, Department of Biomedicine, University Hospital Basel, Basel, Switzerland; 31 Institute of Medical Genetics and Pathology, University Hospital Basel, Basel, Switzerland; 32 Institute of Neuroscience and Medicine (INM-1), Research Center Jülich, Jülich, Germany; 33 Mental Health Research Group, IMIM-Hospital del Mar, Barcelona, Spain; 34 Centro de Investigación Biomédica en Red de Salud Mental (CIBERSAM), Instituto de Salud Carlos III, Madrid, Spain; 35 Douglas Mental Health University Institute, McGill University, Montreal, Canada; 36 Psychiatric Genetic Unit, Poznan University of Medical Sciences, Poznań, Poland; 37 Institute of Human Genetics, University of Bonn, School of Medicine & University Hospital Bonn, Bonn, Germany; 38 Department of Biomedical Sciences, University of Cagliari, Cagliari, Italy; 39 Department of Psychiatry and Behavioral Sciences, Johns Hopkins University, Baltimore, USA; 40 Department of Psychiatry and Psychotherapy, Ludwig-Maximilian-University Munich, Munich, Germany; 41 Department of Adult Psychiatry, Poznan University of Medical Sciences, Poznań, Poland; 42 Department of Genetic Epidemiology in Psychiatry, Central Institute of Mental Health, Medical Faculty Mannheim, University of Heidelberg, Heidelberg, Germany; 43 Centre for Psychiatry Research, Department of Clinical Neuroscience, Karolinska Institutet, Stockholm, Sweden; 44 Neuroscience Research Australia, Sydney, Australia; 45 School of Medical Sciences, University of New South Wales, Sydney, Australia; 46 Laboratorios de Investigación y Desarrollo, Facultad de Ciencias y Filosofía, Universidad Peruana Cayetano Heredia, Porres, Peru; 47 Service de Psychiatrie, Hôpital Charles Perrens, Bordeaux, France; 48 Department of Psychiatry, Dalhousie University, Halifa, Canada; 49 Biometric Psychiatric Genetics Research Unit, Alexandru Obregia Clinical Psychiatric Hospital, Bucharest, Romania; 50 Mood Disorders Center of Ottawa, Ottawa, Canada; 51 Department of Pathology of Mental Diseases, National Institute of Mental Health, National Center of Neurology and Psychiatry, Bunkyo City, Japan; 52 Institute of Psychiatric Phenomics and Genomics (IPPG), University Hospital, LMU Munich, Munich, Germany; 53 Program for Quantitative Genomics, Harvard School of Public Health, Boston, USA; 54 HSL Institute for Aging Research, Harvard Medical School, Boston, USA; 55 Univ Paris Est Créteil, INSERM, IMRB, Translational Neuropsychiatry, Fondation FondaMental, Paris, France; 56 Service de Psychiatrie et Psychologie Clinique, Centre Psychothérapique de Nancy - Université de Lorraine, Paris, France; 57 Department of Psychiatry & Behavioral Science, Juntendo University, Graduate School of Medicine, Bunkyo City, Japan; 58 Department of Psychiatry, Psychosomatic Medicine and Psychotherapy, University Hospital Würzburg, Würzburg, Germany; 59 Department of Public Health & Institute of Epidemiology and Preventive Medicine, College of Public Health, National Taiwan University, Taipei City, Taiwan; 60 Department of Psychiatry, Hokkaido University Graduate School of Medicine, Hokkaido, Japan; 61 Department of Psychiatry and Psychotherapeutic Medicine, Landesklinikum Neunkirchen, Neunkirchen, Austria; 62 Institute of Neuroscience and Physiology, the Sahlgrenska Academy at the Gothenburg University, Göteborg, Sweden; 63 Department of Medical Epidemiology and Biostatistics, Karolinska Institutet, Solna, Sweden; 64 Office of Mental Health, VA San Diego Healthcare System, San Diego, USA; 65 Department of Psychiatry, University of Campania ‘Luigi Vanvitelli’, Caserta, Italy; 66 Section of Psychiatry, Department of Medical Sciences and Public Health, University of Cagliari, Cagliari, Italy; 67 Department of Pharmacology, Dalhousie University, Dalhousie, Canada; 68 Université Paris Cité, Inserm UMR-S 1144, Optimisation Thérapeutique en Neuropsychopharmacologie, F-75006 Paris, France; 69 Department of Clinical Neurosciences, Karolinska Institutet, Karolinska, Sweden; 70 Department of Psychiatry, VA San Diego Healthcare System, San Diego, USA; 71 Department of Psychiatry, Lindner Center of Hope / University of Cincinnati, Ohio, USA; 72 Department of Psychiatry and Psychotherapy, Medical University of Vienna, Vienna, Austria; 73 Comprehensive Center for Clinical Neurosciences and Mental Health, Medical University of Vienna, Vienna, Austria; 74 Department of Genetics, Microbiology and Statistics, Faculty of Biology, University of Barcelona, Barcelona, Spain; 75 Department of Medicine, Surgery and Dentistry ‘Scuola Medica Salernitana’, University of Salerno, Fisciano, Italy; 76 National Institute of Mental Health, Klecany, Czech Republic; 77 Department of Psychiatry & Department of Child and Adolescent Psychiatry, Nagoya University Graduate School of Medicine, Nagoya, Japan; 78 Department of Psychiatry and Psychotherapy, University Hospital Carl Gustav Carus, Medical Faculty, Technische Universität Dresden, Dresden, Germany; 79 Department of Psychiatry, Psychosomatic Medicine and Psychotherapy, University Hospital Frankfurt, Frankfurt, Germany; 80 School of Psychiatry, University of New South Wales, Sydney, Australia; 81 Montreal Neurological Institute and Hospital, McGill University, Montreal, Canada; 82 Northern Adelaide Local Health Network, Mental Health Services, Sydney, Australia; 83 Department of Psychiatry and Psychotherapy, University Hospital Bonn, Medical Faculty University of Bonn, Bonn, Germany; 84 Department of Psychiatry, Dokkyo Medical University School of Medicine, Tochigi, Japan; 85 Bipolar Center Wiener Neustadt, Sigmund Freud University, Medical Faculty, Freudpl, Austria; 86 Epidemiology Branch, Division of Intramural Population Health Research, Eunice Kennedy Shriver National Institute of Child Health and Human Development, National Institutes of Health, Maryland, USA; 87 Department of Psychiatry, University of Perugia, Perugia, Italy; 88 Bipolar and Depressive Disorders Unit, Institute of Neuroscience, Hospital Clinic, University of Barcelona, IDIBAPS, CIBERSAM, ISCIII, Barcelona, Spain; 89 Department of Psychiatry, National Institute of Mental Health and Neurosciences, Bengaluru, India; 90 Department of Mental Health, Johns Hopkins Bloomberg School of Public Health, Baltimore, USA; 91 Department of Psychiatry and Behavioral Sciences, Norton College of Medicine, SUNY Upstate Medical University, Syracuse, New York USA

**Keywords:** Genetics, Bipolar disorder

## Abstract

The link between bipolar disorder (BP) and immune dysfunction remains controversial. While epidemiological studies have long suggested an association, recent research has found only limited evidence of such a relationship. To clarify this, we performed an exploratory study of the contributions of immune-relevant genetic factors to the response to lithium (Li) treatment and the clinical presentation of BP. First, we assessed the association of a large collection of immune-related genes (4925) with Li response, defined by the Retrospective Assessment of the Lithium Response Phenotype Scale (Alda scale), and clinical characteristics in patients with BP from the International Consortium on Lithium Genetics (ConLi^+^Gen, *N* = 2374). Second, we calculated here previously published polygenic scores (PGSs) for immune-related traits and evaluated their associations with Li response and clinical features. Overall, we observed relatively weak associations (*p* < 1 × 10^−4^) with BP phenotypes within immune-related genes. Network and functional enrichment analyses of the top findings from the association analyses of Li response variables showed an overrepresentation of pathways participating in cell adhesion and intercellular communication. These appeared to converge on the well-known Li-induced inhibition of GSK-3β. Association analyses of age-at-onset, number of mood episodes, and presence of psychosis, substance abuse and/or suicidal ideation suggested modest contributions of genes such as *RTN4*, *XKR4*, *NRXN1*, *NRG1/3* and *GRK5* to disease characteristics. PGS analyses returned weak associations (*p* < 0.05) between inflammation markers and the studied BP phenotypes. Our results suggest a modest relationship between immunity and clinical features in BP. More research is needed to assess the potential therapeutic relevance.

## Introduction

Bipolar disorder (BP) has been associated with some degree of immune dysfunction. Epidemiological data has linked immune-related medical comorbidities, including autoimmune and metabolic diseases, and chronic low-grade inflammation with BP. In particular, increases in pro-inflammatory cytokines are observed during affective episodes in patients with BP [1]. In addition, genomic studies have revealed weak yet significant genetic correlation between BP and immune-related diseases [2]. Nevertheless, as a number of these observations originated from underpowered studies [3], further investigations are required to elucidate the proposed relationships.

Lithium (Li), mainly used in the treatment of BP, is an effective pharmacological agent in the treatment of an array of psychiatric conditions [4, 5]. In addition to its mood-stabilizing effects, Li shows anti-viral and immune cell regulatory properties [6, 7]. The immune regulatory activity of Li has been partially attributed to the modulation of pro-inflammatory cytokines and GSK-3β. Therefore, it has been suggested that the mechanism through which Li improves symptom progression may be via anti-inflammatory effects [8, 9]. The Retrospective Assessment of the Lithium Response Phenotype Scale (Alda scale) is the most widely used clinical measure of Li response. Most often, it is dichotomized such that individuals with scores ≥7 are classified as “responders” and those with scores <7 as “non-responders” [10, 11]. Using this metric, previous genetic studies have implicated human leukocyte antigen (HLA) and inflammatory cytokine genes in the response to Li treatment in BP [12, 13]. Therefore, we hypothesized that single nucleotide polymorphisms (SNPs) in immune-related genes contribute, to some extent, to Li response and further, may impact specific clinical features within BP. To test our hypothesis, we performed association studies of a comprehensive collection of immune-related genes in 2374 patients with BP from the International Consortium on Lithium Genetics (ConLi^+^Gen) [14]. Additionally, we tested associations with published polygenic scores (PGSs) for immune-relevant traits.

## Methods

Since our study follows a candidate approach to selected genes, pathways and networks, a diagram summarizing the methodology employed can be found in the Supplementary File 1: Fig. S1.

### Study sample

The ConLi^+^Gen cohort has been previously described in detail [15]. Briefly, peripheral blood samples from individuals with diagnosis of a bipolar spectrum disorder (in accordance with the criteria established in the Diagnostic and Statistical Manual of Mental Disorders—DSM—versions III or IV) that had taken Li for a minimum of 6 months (with no additional mood stabilizers), were collected from 2003 to 2013 at various sites in Europe, the United States, Australia and East Asia. The isolated DNA was genotyped in two phases. This resulted in two sample batches originally referred to as “GWAS1” and “GWAS2”, comprising 1162 and 1401 individuals, respectively. Long-term responses to Li treatment were assessed in both sample batches using the Alda scale. Here, the A subscale rates the degree of response on a 10-point scale, and the B subscale reflects the relationship between improvement and treatment. A total score, ranging from 0–10, is obtained by subtracting the B score from the A score of these subscales. Negative scores are set to 0. Data on age-at-onset (AAO), age (at sample collection and phenotyping), sex and diagnostic subtype were available for both sample batches. Diagnoses included bipolar disorder type I and type II, schizoaffective bipolar disorder and bipolar disorder not otherwise specified. Additionally, information on psychiatric features, namely the number of episodes of depression, mania and hypomania, the presence of psychosis, alcohol and substance abuse, and suicidal ideation, were available for patients in the “GWAS1” batch.

The Ethics Committee at the University of Heidelberg provided central approval for the ConLi^+^Gen Consortium. Written informed consent from all participants was obtained according to the study protocols of each of the participating sites and their institutions. All procedures were performed in accordance with the guidelines of the Declaration of Helsinki.

### Immune gene collection

A comprehensive set of immune-related genes was collated from gene lists available in the online databases MSigDB [16] and InnateDB [17]. From MSigDB (https://www.gsea-msigdb.org/gsea/msigdb/), the following gene sets contained in the C2 “curated gene sets” collection were retrieved: M1036: Reactome-innate immune system, M1058: Reactome-adaptive immune system, M39895: WikiPathways (WP)-neuroinflammation, M39711: WP-cytokines and inflammatory response, and M39641: WP-inflammatory response pathway. From InnateDB (https://www.innatedb.com/index.jsp), the curated gene lists derived from the Immunology Database and Analysis Portal (ImmPort), the Immunogenetic Related Information Source (IRIS) and the Immunome Database, were downloaded. Chromosomal locations were annotated from Ensembl using the hg19 build. Herein, the combined collection is referred to as the ImmuneSet and contained 4925 autosomal genes to be included in association analyses.

### Genotype data

Schubert et al. [18] have previously described the creation of the genotype dataset used herein. Briefly, DNA samples were originally genotyped using either Affymetrix or Illumina SNP arrays. These genotype data from multiple cohorts were separately imputed using the 1000 Genomes Project reference panel phase 3 v5. Each imputed dataset underwent a basic quality control (QC) step to keep variants with minor allele frequency (MAF) > 0.01, Hardy-Weinberg equilibrium *p* value (HWE) ≥ 1 × 10^−6^ and imputation quality score (Rsq) ≥ 0.6. Genotype calls were derived from the imputed dosage scores and all datasets were merged by retaining only common sets of SNPs. To update this dataset and obtain a higher number of good quality variants, we re-imputed the genotype data via the Michigan Imputation Server [19] using the Haplotype Reference Consortium (HRC) panel for European ancestry. The re-imputed genotypes underwent a QC step to keep variants with Rsq ≥ 0.8, MAF ≥ 0.01 and HWE ≥ 1 × 10^−6^. Additionally, individuals were removed if they failed the heterozygosity test and/or showed relatedness, according to the tests performed using the plinkQC R package [20]. In the latter case, one individual from each pair of related individuals (PI-HAT > 0.25) was removed. For analysis of the ImmuneSet, SNPs within each gene’s boundaries (±0 kb) were retained. The final ImmuneSet genotype datasets contained 701,031 SNPs from 1024 and 1350 individuals in “GWAS1” and “GWAS2”, respectively.

### Polygenic scores

A set of 32 published PGSs available at the PGS Catalog [21] were used to approximate markers of inflammation and immune-related phenotypes that were not experimentally measured in the “GWAS1” ConLi^+^Gen sample. These PGSs, created and evaluated in large samples of predominantly European ancestry, stemmed from three recent publications and corresponded to the following traits: autoimmune disease [22], lymphocyte/monocyte/eosinophil/neutrophil/basophil percentage of white (blood) cells [23], and serum levels of 26 markers of inflammation [24]. After downloading and harmonizing weight files, we performed allelic scoring in ConLi^+^Gen using the sum method applied in Plink 1.9 [25]. Sum scores were standardized for statistical analysis.

### Association analyses

The “GWAS1” (*N* = 853) and “GWAS2” (*N* = 1258) samples were tested separately for associations of 701,031 SNPs in the ImmuneSet with: (1) Li response (responder/non-responder, defined by Alda scores ≥7 or <7, respectively), (2) total Alda score, (3) Alda subscale A, and (4) Alda subscale B (total). Because the most reliable continuous Li response phenotype has been previously shown to be the Alda A score, when excluding individuals with Alda B scores >4 [10], we tested this as the primary continuous phenotype in our study. All association tests were performed applying an additive model in Plink 1.9, and all models were adjusted for age at recruitment, age-at-onset (AAO), sex, diagnosis and the first principal components (PCs) obtained for each ImmuneSet genotypes dataset. PCA plots were explored to determine the optimal number of PCs to be used as covariates for each sample. Therefore, the first five PCs were used as covariates for “GWAS1” while the first six PCs were used for “GWAS2”. Population stratification due to ancestry (i.e., European or East Asian) and recruitment site was corrected by the selected numbers of PCs (Supplementary File 1: Fig. S2). Next, the association results for Li response from “GWAS1” and “GWAS2” samples were meta-analyzed using the weighted-z (METAL) method applied in Plink 1.9. These meta-analysis results were QCed to exclude variants with *I*^2^ heterogeneity index (*I*) > 40 and *p* value for Cochran’s *Q* statistic (*Q*) < 0.1 (highly heterogeneous). We searched first for associations at the commonly accepted thresholds for GWASs (genome-wide association, *p* < 5 × 10^−8^, and suggestive association, *p* < 1 × 10^−5^). However, considering this a candidate gene rather than a genome-wide approach, we chose to look further into findings with *p* < 1 × 10^−4^, a threshold that has been previously used to select association findings for follow-up in GWASs [26] and, therefore, represents an acceptable exploratory threshold.

In “GWAS1”, the ImmuneSet was further tested for associations with other BP clinical phenotypes (i.e., AAO, the number of episodes of depression, mania and hypomania, as well as the presence of psychosis, alcohol and/or substance abuse, and suicidal ideation). These models were adjusted for age at recruitment, AAO (except when AAO was tested as phenotype), sex, diagnosis and the first five PCs. Statistical association and exploratory thresholds were considered as above.

Associations between PGSs and the various BP clinical phenotypes were tested using linear or binomial regression models, as appropriate, adjusted for age at recruitment, AAO (except when AAO was tested as phenotype), sex, diagnosis, recruitment site and the first 10 PCs obtained from the genotypes using the robustbase R package. Statistical association was set to false discovery rate (FDR) < 0.05. The exploratory threshold was set to *p* < 0.05.

### Downstream analyses

All variants under the *p* < 1 × 10^−4^ threshold were annotated for known regulatory effects on gene expression (i.e., expression quantitative trait loci, eQTLs) in all human brain, blood, spleen and thyroid tissues, as well as in immune cells (e.g., monocytes and macrophages) using Qtlizer [27].

A protein-protein interaction (PPI) network to investigate the functional relevance of the genes linked to Li response by the exploratory analyses was created using the ReactomeFIViz app [28] for Cytoscape 3.7 [29]. This analysis used as input a list composed of the ImmuneSet genes showing associations at the *p* < 1 × 10^−4^ threshold with the dichotomous and continuous Li response phenotypes. The network also incorporated “linker” genes (i.e., genes not in the input gene list that create indirect connections between input genes) to increase biological interpretability. Moreover, pathway overrepresentation analysis was performed on the PPI network (including linker genes) using the pathway enrichment network function of the app. Because the linker genes were not drawn from the ImmuneSet collection, we used the standard background genes of the ReactomeFiViz app for this analysis. The resulting overrepresented pathways were filtered to exclude terms that: (1) had FDR > 0.05, (2) corresponded to a specific disease (e.g., bladder cancer, herpes virus infection), (3) had less than two genes overlapping between the pathway set and the network set, and/or (4) the overlap with the pathway set represented less than 3% of genes in the set. Additionally, we repeated the pathway overrepresentation analysis including not only the variant mapped genes, but also the annotated eQTL genes.

For associations with clinical phenotypes in the “GWAS1” sample, functional analyses were performed using the GENE2FUNC tool of the Functional Mapping and Annotation of Genome-Wide Association Studies (FUMA-GWAS) platform [30]. The input gene lists included mapped and eQTL genes annotated for variants below the *p* < 1 × 10^−4^ threshold for each studied phenotype. Because eQTL genes were not drawn from our ImmuneSet collection, we used all protein-coding genes as background for these analyses. Overrepresented gene sets were those that showed FDR < 0.05, following a hypergeometric test, and a minimum of two overlapping genes. Curated gene sets from pathway databases in the “canonical pathways” category were preferred when available. Otherwise, Gene Ontology biological processes (GO_BPs) or any other available category (including GWAS Catalog trait associations) were taken. For GTEx-based enriched tissues of expression, as our focus is on immune-brain relationships, we kept only those enrichments corresponding to brain expression, as these are the most relevant tissues for the analysis of Li response and clinical features of BP.

In addition, the relative importance for (dichotomous) Li response of the calculated PGSs in “GWAS1” was assessed through a machine learning (ML) screening approach using the Auto Model extension of RapidMiner Studio. This applied various classification algorithms to the raw PGS data. Auto Model provides the following models: Naïve Bayes, Generalized Linear Model, Logistic Regression, Fast Large Margin, Deep Learning, Decision Tree, Random Forest, Gradient Boosted Trees and Support Vector Machines. Because ML algorithms are sensitive to class imbalance, an equal number of responder and non-responder individuals were randomly selected for the analysis (*N* = 657) using the sample method of the Python’s Pandas library. The resulting file with balanced classes was used as input for the ML screening in Auto Model using default parameters for all algorithms, which included split validation with stratified sampling, where the sample was randomly divided into training (60%) and test (40%) sets. Given that different types of ML algorithms can differ in their feature selection procedure due to their inherent characteristics, here, features were considered important for Li response, with either a positive (i.e., favoring response) or a negative (i.e., favoring non-response) effect, when at least two algorithms selected the same feature with the same effect direction as important for the classification task.

## Results

After excluding individuals with missing phenotypic data (age and/or AAO), the effective sample sizes for the association analyses in ConLi^+^Gen were 853 and 1258 in “GWAS1” and “GWAS2”, respectively. A basic description of both samples is shown in Table 1. In general, there were more female than male patients in both samples and there were minimal differences in the mean ages at recruitment and disease onset between “GWAS1” and “GWAS2”. Therefore, the total sample size of our meta-analyses of Li response was 2111, including 606 (28.7%) responders and 1505 non-responders for the dichotomized variable, which included 1224 (58%) females and 887 males, with mean age 47 (±14) years and mean AAO 25 (±11) years. For the continuous Li response phenotype (i.e., Alda A score, excluding individuals with Alda B score >4), the effective sample sizes were 828 for “GWAS1” and 1044 for “GWAS2”. After the post-meta-analysis exclusion of variants with heterogeneous effects between both ConLi^+^Gen samples, a mean of 556,196 SNPs remained in each set of summary statistics. This was higher for the continuous phenotype, in which 625,818 SNPs remained.

**Table 1:** Description of the ConLi^+^Gen samples.

	Responders (Total Alda ≥ 7)	Non-responders (Total Alda < 7)	Total
GWAS1			
*N* Effective sample (% from total)	297 (34.8)	556 (65.2)	853
*N* Females (%)	178 (59.9)	337 (60.6)	515 (60.4)
Age (mean ± SD)	52 ± 14	46 ± 14	48 ± 14
Age-at-onset (mean ± SD)	28 ± 11	23 ± 11	25 ± 11
# Depressive episodes (mean)	5	7	6
# Hypomanic episodes (mean)	2	6	4
# Manic episodes (mean)	4	6	5
*N* Psychosis cases (%)	72 (24.2)	270 (48.6)	342 (40.1)
*N* Alcohol abuse cases (%)	18 (6.1)	122 (21.9)	140 (16.4)
*N* Substance abuse cases (%)	30 (10.1)	105 (18.9)	135 (15.8)
*N* Suicidal ideation cases (%)	75 (25.3)	256 (46.0)	331 (38.8)
GWAS2			
*N* Effective sample (% from total)	309 (24.6)	949 (75.4)	1258
Females (%)	157 (50.8)	552 (58.2)	709 (56.4)
Age (mean ± SD)	48 ± 15	46 ± 13	47 ± 14
Age-at-onset (mean ± SD)	25 ± 10	25 ± 11	25 ± 11

### Immune-related genes showed modest associations with response to Li treatment in BP

We found no associations with Li response at the genome-wide GWAS threshold (*p* < 5 × 10^−8^). At the suggestive threshold for GWAS (*p* < 1 × 10^−5^), the dichotomous Li response phenotype and Alda B (total) showed associations with *FAT3* (best SNP: rs4313539, *p* = 2.1 × 10^−6^, *z* = 4.744), and with *ADAMTS5* (best SNP: rs162501, *p* = 9.18 × 10^−7^, *z* = 4.909) and *GRID2* (best SNP: rs62312225, *p* = 2.71 × 10^−6^, *z* = 4.692), respectively (Supplementary File 2: Table 1).

At the exploratory threshold (*p* < 1 × 10^−4^), when considering linkage disequilibrium (LD), we identified between 9 and 12 genomic loci in relation with different aspects of Li response (Supplementary File 2: Table 1). The top SNPs from the analyses of the dichotomous and continuous phenotypes mapped to *FAT3* and *BMPR1A*, respectively (Table 2). In total, 42 genes were linked to the response to Li in patients with BP from our exploratory analyses in the ConLi^+^Gen cohort (Supplementary File 3: Table 1). There was a number of gene-based overlaps between different aspects of Li response, particularly between the dichotomous variable and total Alda score, and between the continuous variable and the other Alda variables.

**Table 2:** Summary findings from the genetic association meta-analyses of Li responses in ConLi^+^Gen.

Meta-analysis summary	Response vs. No-response	Continuous Li response
# SNPs after QC	557,037	625,818
# SNPs *p* < 0.05	27,426	31,489
# SNPs *p* < 1 × 10^−4^	124	33
# Lead SNPs	11	11
Top lead SNP	rs4313539	rs12776537
Effect allele	C	A
*p* value	2.1 × 10^−6^	2.5 × 10^−5^
*Z*	4.7	−4.2
Gene	*FAT3*	*BMPR1A*
# SNPs *p* < 1 × 10^−5^	15	0

**Fig. 1:**
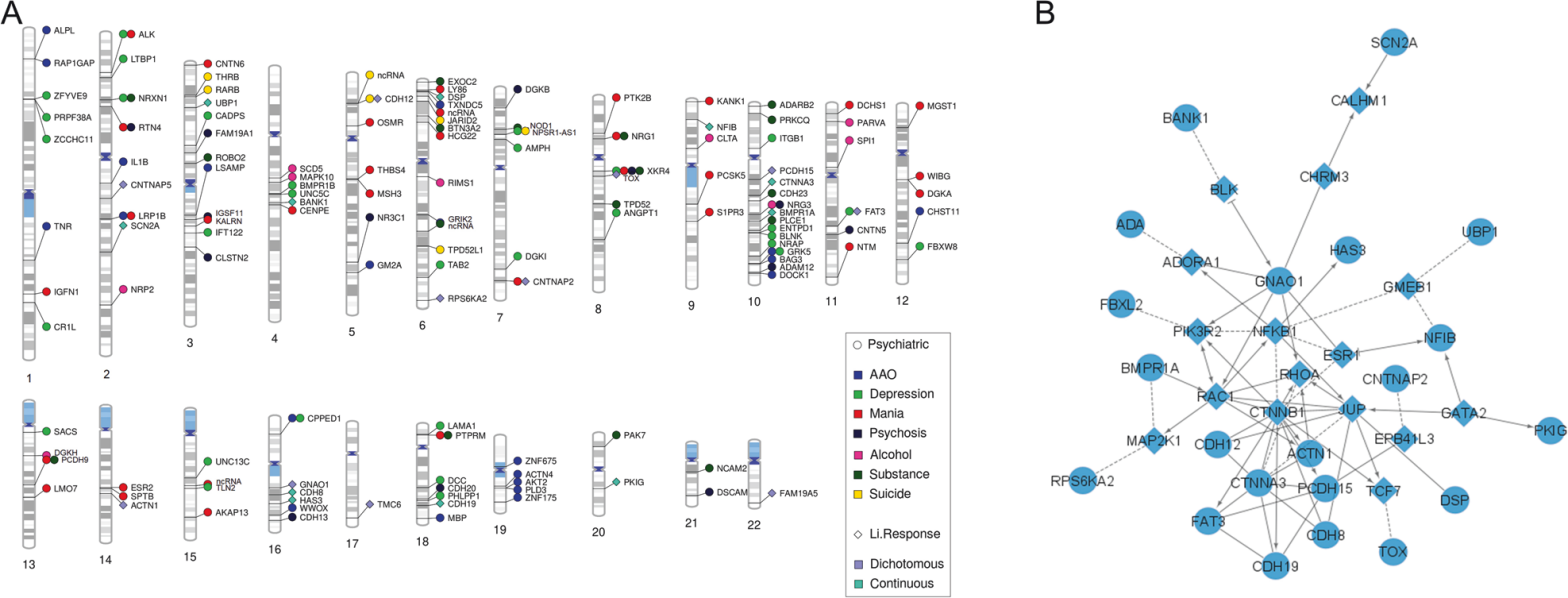
Exploratory findings for the ImmuneSet in ConLi^+^Gen. **A** Gene-based phenogram of associations of the ImmuneSet with Li response and clinical features in ConLi^+^Gen. **B** Protein-protein interaction network of Li response phenotype associations in the ImmuneSet. Circles represent input genes and diamonds represent linker genes. Dotted lines denote predicted interactions.

**Fig. 2:**
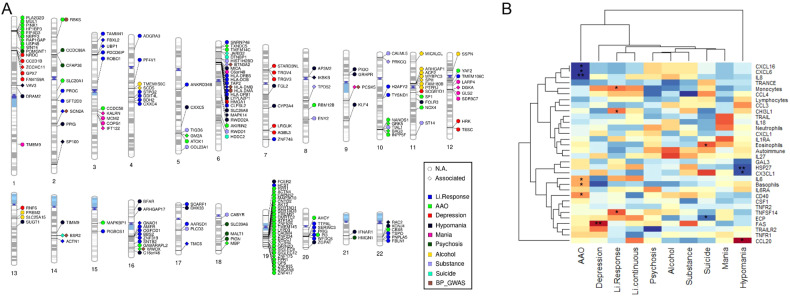
Results of eQTL annotation and PGS association testing. **A** Gene-based phenogram of eQTL annotations for the exploratory-level findings of the ImmuneSet in ConLi^+^Gen. Those eQTL genes that were different from the mapped gene and those that were the same are presented in circle and diamond shapes, respectively. Only protein-coding genes are shown. In addition, overlaps with BP genetic associations reported in the GWAS Catalog are presented. **B** Exploratory findings from the association analyses of immune-related polygenic scores and BP phenotypes in ConLi^+^Gen “GWAS1”. The heatmap shows the estimates (scaled by column) obtained for each PGS-phenotype pair. Increasing color darkness alludes to increasing effect, with red and blue colors representing positive and negative values, respectively. *p < 0.05, **p < 0.01.

Twenty-four ImmuneSet genes were linked to the primary Li response phenotypes (i.e., dichotomous and continuous) in our exploratory analyses (Fig. 1A). These were used as input for a network analysis to facilitate biological interpretation of the findings. This network analysis provided known and predicted functional interactions between a subset of 21 input genes from our exploratory association results and 16 linkers drawn from the total of protein-coding genes in the background reference of the ReactomeFiViz app (Fig. 1B). Functional analysis of the network showed an overrepresentation (FDR < 0.05) of crucial developmental pathways and regulatory networks, including processes such as assembly and stability of the cell-cell signaling machinery (e.g., adherens junction, E-cadherin signaling, focal adhesion, integrin signaling, L1 cell adhesion molecule signaling), neuronal development and function (e.g., neurotrophic signaling, lysophosphatidic acid receptor mediated events, regulation of pluripotency, Wnt signaling), as well as activation of inflammatory (e.g., sphingolipid signaling, S1P pathways, toll-like receptor signaling) and adaptive immune pathways (e.g., T and B cell receptor signaling). Interestingly, processes such as angiogenesis, long-term potentiation, thyroid hormone signaling pathways, melanogenesis and sensory processing were also overrepresented in our network analysis (Supplementary File 3: Table 2). When eQTL genes were incorporated into the analysis, there was a marked overrepresentation of inflammatory and autoimmune disease pathways (e.g., asthma, type 1 diabetes mellitus, autoimmune thyroid disease, inflammatory bowel disease), and of vitamin D metabolism. The Wnt signaling, cell adhesion and adaptive immune pathways remained overrepresented (data not shown). All protein-coding eQTL genes annotated for Li response phenotypes are shown in Fig. 2A.

### Immune-related genes showed modest associations with clinical phenotypes in BP

The association analyses of the ImmuneSet with specific clinical features that were available for the “GWAS1” sample showed no associations at the genome-wide GWAS threshold. However, at the suggestive GWAS threshold there were, collectively, 100 associations between the ImmuneSet and AAO (3), number of depressive (54) and manic (14) episodes, and the presence of psychosis (1), substance use disorder (15) and/or suicidal ideation (13). These covered 17 genes that associated to specific clinical features (i.e., no overlaps were observed at this threshold; Table 3).

**Table 3:** Summary of findings from the association analyses of the ImmuneSet with clinical characteristics in the ConLi^+^Gen “GWAS1” sample.

Phenotype	*N*	Exploratory threshold (*p* < 1e−4)		Suggestive GWAS threshold (*p* < 1e−5)			Top gene	
		# SNPs	# Genes	# SNPs	# Genes	Genes	Symbol	*p* val
Age-at-onset	853	54	21	3	2	*GRK5, PLD3*	*GRK5*	3.9 × 10^−6^
# Depressive episodes	692	107	31	54	6	*BLNK, PHLPP1, ZCCHC11, SACS, CPPED1, PRPF38A*	*BLNK*	1.7 × 10^−7^
# Manic episodes	665	116	32	14	4	*CNTN6, KALRN, LY86, PTK2B*	*CNTN6*	2.4 × 10^−6^
Psychosis	692	45	13	1	1	*DSCAM*	*DSCAM*	7.6 × 10^−6^
Alcohol abuse	835	29	9	0	0	*–*	*SCD5*	1.8 × 10^−5^
Substance abuse	832	78	17	15	3	*TPD52, NOD1, XKR4*	*TPD52*	4.5 x 10^−7^
Suicidal ideation	660	30	7	13	1	*JARID2*	*JARID2*	3.9 × 10^−6^

When we moved forward to the exploratory analysis, we identified 786 SNP-phenotype associations in total (Supplementary File 2: Tables 2–9). These involved 166 immune-related genes (Fig. 1A and Supplementary File 3: Table 1) mostly involved in adaptive immunity and inflammation. Beyond their immune functions, according to our functional analyses, these genes play important roles in the development of the nervous system, signal transduction, synaptic processes and cell adhesion (Supplementary File 3: Tables 3–9). In particular, large numbers of associations were suggested for AAO and mood episodes (Table 3). In addition, 156 eQTL genes were collectively annotated for these phenotypes. Figure 2A shows the protein-coding eQTL genes annotated for each clinical feature. These showed no overlaps among the clinical phenotypes. Nevertheless, there were some overlaps with the ImmuneSet genes given that, in some instances, the eQTL gene corresponded to the gene mapped to the variant while, in others, the eQTL gene was different from the mapped gene. A summary of exploratory findings for each clinical phenotype studied can be found in the Supplementary File 1: Supplementary Results.

### Immune-related genes showed pleiotropy for BP phenotypes

A number of genes showed shared associations with the different phenotypes included in our exploratory ImmuneSet analyses in ConLi^+^Gen (Fig. 1A). These genes can be prioritized for follow-up studies due to their pleiotropic effects in Li response, clinical features, or both. In this way, we prioritized 21 genes linked with more than one BP phenotype in our exploratory analyses (Table 4). Here, we excluded genes linked to Alda A, total Alda B and/or Total Alda when there was no overlap with the dichotomous and/or continuous Li response phenotypes. However, a complete list of corresponding gene-phenotype exploratory findings (197 in total) is shown in Supplementary File 3: Table 1. Five of the prioritized genes (*CNTNAP5*, *DSP*, *NFIB, BMPR1A*, and *HAS3*) were associated with multiple Li response phenotypes, 10 of them (*XKR4*, *NRXN1*, *RTN4*, *NRG1/3*, *ALK, GRK5, LRP1B, NPSR1-AS1* and *CPPED1*) were associated with multiple clinical features, and another six genes (*BANK1*, *ROBO2*, *CNTNAP2*, *PCDH9, CDH12* and *FAT3*) were associated with Li response as well as with clinical features.

**Table 4:** Prioritized ImmuneSet candidate genes for Li response and clinical characteristics in ConLi^+^Gen.

Gene	Chr	Start	End	Priority	Phenotypes
*ALK*	2	29415640	30144432	Psychiatric	Depression, Mania
*NRXN1*	2	50145643	51259674	Psychiatric	Depression, Hypomania, Substance
*RTN4*	2	55199325	55339757	Psychiatric	Mania, Psychosis
*CNTNAP5*	2	124025287	124915287	Li response	LiResponse, Alda_B, Alda_Total
*LRP1B*	2	140988992	142889270	Psychiatric	AAO, Mania
*ROBO2*	3	75906695	77649964	Both	Alda_Total, Hypomania, Substance
*BANK1*	4	101411286	102074812	Both	Continuous.LiResp, Alda_A, Alda_B, Alda_Total, Hypomania
*CDH12*	5	21750673	22853622	Both	LiResponse, Suicide
*DSP*	6	7541575	7586717	Li response	Continuous.LiResp, Alda_Total
*NPSR1-AS1*	7	34386124	34911194	Psychiatric	Depression, Suicide
*CNTNAP2*	7	146116002	148420998	Both	LiResponse, Alda_Total, Mania
*NRG1*	8	31496902	32622548	Psychiatric	Mania, Substance
*XKR4*	8	56014949	56454613	Psychiatric	Depression, Mania, Psychosis, Substance
*NFIB*	9	14081843	14398983	Li response	Continuous.LiResp, Alda_B
*NRG3*	10	83635070	84746935	Psychiatric	Alcohol, Psychosis
*BMPR1A*	10	86756601	86932838	Li response	Continuous.LiResp, Alda_A
*GRK5*	10	120967101	121215131	Psychiatric	AAO, Depression
*FAT3*	11	92352096	92896470	Both	LiResponse, Alda_Total, Depression
*PCDH9*	13	66302834	67230445	Both	Alda_B, Mania, Substance
*CPPED1*	16	12756919	12897874	Psychiatric	AAO, Depression
*HAS3*	16	69105564	69118719	Li response	Continuous.LiResp, Alda_A, Alda_Total

### Polygenic scores for immune-related traits weakly associated with BP phenotypes

In addition to testing associations of the ImmuneSet with BP phenotypes, we calculated a set of 32 previously published immune-related PGSs, namely for: (1) (general) autoimmune disease, (2) the proportions of white blood cell populations and (3) inflammatory marker levels in serum (Supplementary File 3: Table 10). The overlap of variants between the PGS weight files obtained from PGS Catalog and the SNPs available in our ConLi^+^Gen “GWAS1” sample was, in general, better for the serum levels of inflammatory markers (80.6% in average) than for the other PGSs. The lowest valid SNP overlap was observed for the PGS of general autoimmune disease (38.8%). For the proportion of white blood cell populations, the valid SNP overlap was also not fully satisfactory (49.4% in average). Results from these analyses should be interpreted with caution, as the calculated PGSs may poorly index autoimmune disease and the proportion of white blood cell signatures.

We identified 15 PGS nominal (*p* < 0.05) associations with BP phenotypes (Fig. 2B): six with AAO (basophils, CD40, CXCL6/16, IL-6/8), three with the number of episodes of hypomania (CCL20, CX3CL1, HSP27) and Li response (monocytes, CHI3L1, TNFSF14), two with the presence of suicidal ideation (eosinophils, ECP), and one with the number of depressive episodes (FAS). However, none of these survived correction for multiple comparisons (Supplementary File 3: Table 10).

Finally, ML-based PGS ranking for the dichotomized Li response suggested relative importance of serum markers of inflammation such as ECP, HSP27, CHI3L1, TRAILR2 and TNFSF14 for the prediction of responses to Li treatment in ConLi^+^Gen (Supplementary File 1: Fig. S3).

## Discussion

There is apparent mounting evidence of immune dysregulation in BP and other major psychiatric diseases. Nevertheless, some observations have originated from underpowered studies, resulting in a lack of reproducibility [3]. Therefore, it becomes crucial to gain a better understanding of the relationships between the immune and central nervous systems, and to discern between causes and consequences of disease. With this in mind, we sought to investigate how genetic factors relevant to immune activity relate to disease phenotypes, such as response to Li treatment, AAO and psychiatric symptoms, in patients with BP. Using an exploratory and extensive candidate gene approach, our study suggested various genes and inflammatory markers that appeared to represent potential pleiotropic factors with modest contributions to multiple BP phenotypes. However, we should note that, here, we refer to pleiotropy as the (suggested) association with multiple traits in the ConLi^+^Gen cohort and, by no means, have we implied that these features are independent from each other. In fact, it should be expected that, given the important correlation between psychiatric disorders, the features that we have studied in ConLi^+^Gen are, to some extent, also correlated with each other. Additionally, because genes can play different roles in different tissues and cell types, we observed widespread enrichments of biological pathways participating in the development and function of the brain. This suggested that the genes contributing to shape BP phenotypes might affect in parallel both the immune and nervous systems. Nevertheless, because we found no associations at the genome-wide GWAS threshold, and those observed at the suggestive GWAS threshold were limited, our findings are also consistent with a relatively weak effect of immune genetic factors over BP phenotypes.

The results of our exploratory assessment of associations of genetic polymorphisms in immune-related genes with Li response in the ConLi^+^Gen cohort suggest that variations in multiple inflammatory and adaptive immune processes might modestly contribute to the response to Li treatment in patients with BP. Importantly, our network and gene set enrichment analyses localized these modest contributions to numerous biological pathways that participate in cell adhesion, migration and intercellular communication, which help in the development and maintenance of the central and peripheral nervous systems, as well as of the immune and vascular systems. Interestingly, many of these processes appear to converge in the participation of GSK-3β (glycogen synthase kinase-3 beta), as assessed through comparative overlap analysis of the enriched KEGG and Reactome gene sets. GSK-3β is involved in multiple major developmental pathways, such as the Wnt, Notch and Hedgehog signaling pathways. Genetic manipulation in mouse models has shown an antidepressant-like behavior upon GSK-3β knockdown in hippocampus, as well as cognitive, behavioral and biochemical changes associated with psychiatric disorders, including Alzheimer’s disease, BP and schizophrenia, upon GSK-3β overexpression [31]. Li possesses a well-known inhibitory effect over GSK-3β [32]. Therefore, our analyses suggest that GSK-3β might be a relevant player in the biological response to Li treatment in BP. Our findings are also in agreement with other epidemiological and molecular investigations of Li effects. For example, we observed overrepresentation of various gene sets related to thyroid function, such as thyroid-stimulating hormone signaling and autoimmune thyroid disease. This is in line with the reports of a reversible association of Li treatment with hypothyroidism, particularly in women [33, 34].

Taken together, the encouraging literature supporting our exploratory findings sparked our interest in evaluating how a genetic measure (PGS) of immune-relevant traits, such as inflammatory marker levels in serum and the proportion of white blood cells, might associate with Li response in ConLi^+^Gen. Here, we observed only weak statistical associations. However, one might consider these results inconclusive given the limited overlap between PGS weight variants and our ConLi^+^Gen dataset. Moreover, it must be kept in mind that PGSs have an incomplete indexing of the trait and, in this case, do not reflect the real levels of inflammation markers present in the serum of these patients. Considering this, we adopted a different approach to explore the relationship between PGSs and the dichotomous Li response in ConLi^+^Gen. Here, we used RapidMiner’s Auto Model to probe various ML algorithms and extract the relative importance of each PGS to predict Li response. Given the PGS complications mentioned above and the well-known fitting issues arising from the use of small sample sizes in predictive modeling, we expected a relatively poor overall performance of the PGS model and, hence, we were not looking to evaluate predictive value. Indeed, the accuracy of the tested algorithms was only 43–56% (data not shown). These observations should be interpreted with caution due to the relatively low robustness of split validation in assessing model performance compared to other validation approaches used in predictive ML. Nevertheless, applied for feature importance ranking, this ML approach had the advantage of providing information on the PGS relationships with Li response when compared to one another.

The results of our exploratory assessment of associations of genetic polymorphisms in the ImmuneSet genes with BP’s AAO, numbers of mood episodes and psychiatric comorbidities in ConLi^+^Gen identified various genes potentially contributing to mood episodes and substance abuse, including *XKR4*, *NRXN1*, *GRK5* and *NRG1/3*. These genes, besides their immune-related functionalities, seem to play important roles in neuronal development and function, according to our gene set enrichment analyses. Indeed, this could be corroborated by the literature in many instances. For example, *NRXN1*, a cell surface protein involved in cell-cell interactions, exocytosis of secretory granules and regulation of signal transmission, has been associated with autism, schizophrenia and nicotine dependence [35]. *GRK5* has a role in the regulation of motility in polymorphonuclear leukocytes and inflammation [36, 37]. It also regulates the activity of various G-protein coupled receptors, including neurotransmitter receptors [38]. *XKR4*, a phospholipid scramblase strongly expressed in brain tissue and activated by caspases, has been suggested to participate in the remodeling of neural networks by triggering microglial responses to the exposure of phosphatidylserine on axons, dendrites and synapses [39].

It is worth noting that three of our potential candidate genes for depressive episodes have previously shown associations with either major depression at the gene-level (*DCC*) [40] or mapped to schizophrenia loci (*CR1L* and *DGKI*) [41] in large GWASs. Moreover, one of our suggested candidates for manic episodes, *ESR2*, and one for substance use disorder, *BTN3A2*, were previously associated at the gene-level with major depression in the large GWAS, while one of our candidates for hypomanic episodes and alcohol dependence, the BP-associated *RIMS1* [26], mapped previously to schizophrenia loci. This, together with the overlaps with reported BP-associated genes that we observed, particularly concerning known eQTL genes, provides some support to the validity of our exploratory findings.

Finally, even when our assessment of PGS associations with clinical features resulted only in nominal associations that did not survive correction for multiple testing, we might (cautiously) draw a few interesting observations and new hypotheses for further exploration. For example, that activation of macrophages and neutrophils, reflected by the potential links with the PGSs for cytokines and chemokines produced by or targeting these cells (e.g., interleukin-6/8, CXCL6/16), might contribute to disease AAO in ConLi^+^Gen. These observations might be supported by studies that found increases in neutrophil counts in psychiatric disorders, including BP [42, 43], as well as association of genetic polymorphisms in interleukin-1β, a pro-inflammatory cytokine produced by activated immune cells, including neutrophils and macrophages, with age of onset of depression in geriatric patients [44]. In addition, if we consider that macrophage/neutrophil activation was also a suggested mechanism of Li response in our study, it would be easy to speculate that activation of these cells might be linked with some aspect of the disease onset.

In conclusion, we performed an exploratory study that suggests a modest relationship between immunity and clinically relevant BP phenotypes at the genetic level and identified various interesting potential candidates for follow-up studies. We acknowledge that our study was limited by a relatively small sample size, particularly for the episodes of hypomania, and by incomplete overlap between the variants in the PGSs and our ConLi^+^Gen dataset, likely resulting from a limited overlap among the different SNP arrays initially used to genotype samples in different collection centers. Moreover, although regulatory regions were out of the scope of our study due to their high complexity for interpretation, we acknowledge that restricting our analyses to the gene’s boundaries excluded potentially interesting regulatory SNPs involved in Li response and BP characteristics. Finally, aware that the inclusion of East Asian subsamples in our genetic analyses might rise some concerns, we performed a sensitivity analysis by excluding East Asian individuals from the genetic association tests of primary Li response phenotypes (data not shown). The QCed meta-analysis results of both dichotomous (Pearson *r* = 0.975) and continuous (Pearson *r* = 0.941) Li response variables performed with and without East Asian samples were highly correlated. Because we expected mild differences between analyses due to the loss in sample size, these results suggested that any potential bias that could be attributed to the different ancestries was addressed in our analyses. Despite the inherent limitations and the modest significance of our findings, we believe that our study provides plausible biological insights that might further the understanding of immune contributions to BP. Further studies are needed to elucidate if and how immune regulation might represent a feasible strategy to improve the symptomatology and treatment response in patients with BP.

### Supplementary information

Supplementary File 1

Supplementary File 2

Supplementary File 3

## Data Availability

The data that support the findings of this study are available from ConLi^+^Gen, but restrictions apply to their availability.
