# Supplementary material for: Immunogenetics of lithium response and psychiatric phenotypes in patients with bipolar disorder
Source: Transl Psychiatry. 2024 Apr 3;14:174. doi: 10.1038/s41398-024-02865-4 (PMC10991481; doi:10.1038/s41398-024-02865-4)
Supplement: Supplementary file 1 [file 41398_2024_2865_MOESM1_ESM.pdf]

Immunogenetics of lithium response and psychiatric phenotypes in patients with bipolar disorder

Supplementary Results

Age-at-onset. Fifty-four associations in 21 ImmuneSet genes were found for AAO in our study (Table 3, Supplementary File 2: Table 2). These genes were enriched for negative regulation of cell death and synaptic transmission, as well as expression in the cerebellum (Supplementary File 3: Table 3). The top variant, rs1248079 ($p=3.9x10^{-6}$, beta=2.75), mapped to an intronic region in *GRK5* (G Protein-Coupled Receptor Kinase 5). Other important genes included *PLD3*, *AKT2* and *IL1B*. The addition of eQTL genes to the functional enrichment analysis resulted in an additional overrepresentation of processes related to cellular stress responses.

Depression. With an effective sample of 692 individuals, 107 associations in 31 ImmuneSet genes for the number of depressive episodes were found (Table 3, Supplementary File 2: Table 3). These genes were enriched for synaptic processes, as well as expression in frontal and anterior cingulate cortices (Supplementary File 3: Table 4). While the top variant, rs55975329 ($p=1.7x10^{-7}$, beta=4.54), localized to an intron in *BLNK* (B cell linker), other important genes included *PHLPP1*, *ZCCHC11 (TUT4)* and *CPPED1*. The addition of eQTL genes to the functional enrichment analysis resulted in an additional overrepresentation of axonal and synaptic components.

Hypomania. Although the largest associations were found for the number of hypomanic episodes, these observations were based on only 85 individuals with available data. Therefore, we have excluded this phenotype from the figures and tables shown within this manuscript. All corresponding results are provided in the supplementary material (Supplementary File 2: Table 4, Supplementary File 3: Table 5).

Mania. With an effective sample of 665 individuals, 116 associations in 32 ImmuneSet genes for the number of manic episodes were found (Table 3, Supplementary File 2: Table 5). These genes were enriched for neuronal development and differentiation, as well as expression in spinal cord and frontal cortex (Supplementary File 3: Table 6). The top variant, rs59134172 ($p=2.4x10^{-6}$, beta=1.62), localized to an intron in *CNTN6* (Contactin 6). Other important genes included *KALRN*, *PCDH9, PTK2B* and *CNTNAP2*. Interestingly, we also found enrichment for response to serotonin re-uptake inhibitors in major depressive disorder (FDR=0.042) and serum thyroid-stimulating hormone levels (FDR=0.0028), from the GWAS Catalog trait associations, in mania-associated ImmuneSet genes. The addition of eQTL genes to the functional enrichment analysis had no impact on the overrepresented gene set classes.

Psychosis. The effective sample for our analysis of the presence of psychosis in BP was 692 individuals. Here, 45 associations in 13 genes were identified (Table 3, Supplementary File 2: Table 6). The implicated genes were enriched for cell adhesion and synapse organization, as well as expression in frontal cortex (Supplementary File 3: Table 7). The top SNP, rs459374 ($p=7.6x10^{-6}$, beta=1.96), is located in an intronic region of *DSCAM* (DS Cell Adhesion Molecule). Other interesting genes included *CLSTN2*, *RTN4* and *CDH13*. Moreover, the GWAS Catalog traits seasonality and depression (FDR=0.02), and response to amphetamines (FDR=0.026), as well as obesity-related traits (FDR=0.015), atrial fibrillation (FDR=0.027) and diastolic blood pressure (FDR=0.027) were enriched among the psychosis-associated genes.

The addition of eQTL genes to the functional enrichment analysis had no impact on the overrepresented processes. However, this resulted in a considerable increase in overrepresented brain tissues of expression, including the hippocampus, amygdala, hipothalamus, anterior cingulate cortex, putamen and substantia nigra.

Alcohol and substance abuse. The effective sample sizes for alcohol and substance use disorders in ConLi$^+$Gen were 835 and 832, respectively. Twenty-nine SNPs in nine genes were associated with alcohol use, with the rs7698751 SNP in *SCD5* (Stearoyl-CoA Desaturase 5) being the top association (p=1.8x10^{-5}, beta=2.61). Although no gene set enrichments were found for associations with alcohol abuse, other implicated genes included the BP-associated *DGKH, NRG3* and *RIMS1* (Supplementary File 2: Table 7). Interestingly, when incorporating the eQTLs genes into this functional analysis, overrepresentation of genes associated with mood swings, loneliness and anxious behaviors in the GWAS Catalog was observed (Supplementary File 3: Table 8). For substance use disorder, 78 associations implicating 17 genes were found (Supplementary File 2: Table 8). Genes were overrepresented in neurogenesis-related processes, with expression in cerebellum as well as frontal and anterior cingulate cortices (Supplementary File 3: Table 8). The top variant, rs7814474 (p=4.5x10^{-7}, beta=6.7), was mapped to an intron in *TPD52* (Tumor Protein D52). Other genes included the BP-associated *NRG1*, the schizophrenia-associated *PTPRM*, as well as *NOD1* and *PRKCQ*. In addition, the GWAS Catalog traits chronotype (FDR=0.011) and serum thyroid-stimulating hormone levels (FDR=0.047) were also overrepresented in substance abuse-associated ImmuneSet genes. The addition of eQTL genes to the functional enrichment analysis resulted in an additional overrepresentation of the phosphatidylinositol signaling system and expression in the amygdala, hippocampus and basal ganglia.

Suicidal ideation. Information on the presence of suicidal thoughts was available for 660 ConLi$^+$Gen individuals. Based on these, 30 variants in seven genes were associated with suicidal ideation in the "GWAS1" sample (Table 3, Supplementary File 2: Table 9). The top SNP was rs2327882 (p=3.9x10^{-6}, beta=0.55), located in an intron of the *JARID2* (Jumonji and AT-Rich Interaction Domain Containing 2) gene. Gene set enrichment analysis found overrepresentation of functions of nuclear receptors (Supplementary File 3: Table 9). Indeed, these terms related to *RARB* and *THRB*. The addition of eQTL genes to the functional enrichment analysis had no impact on the overrepresented gene set classes.

Supplementary Figures

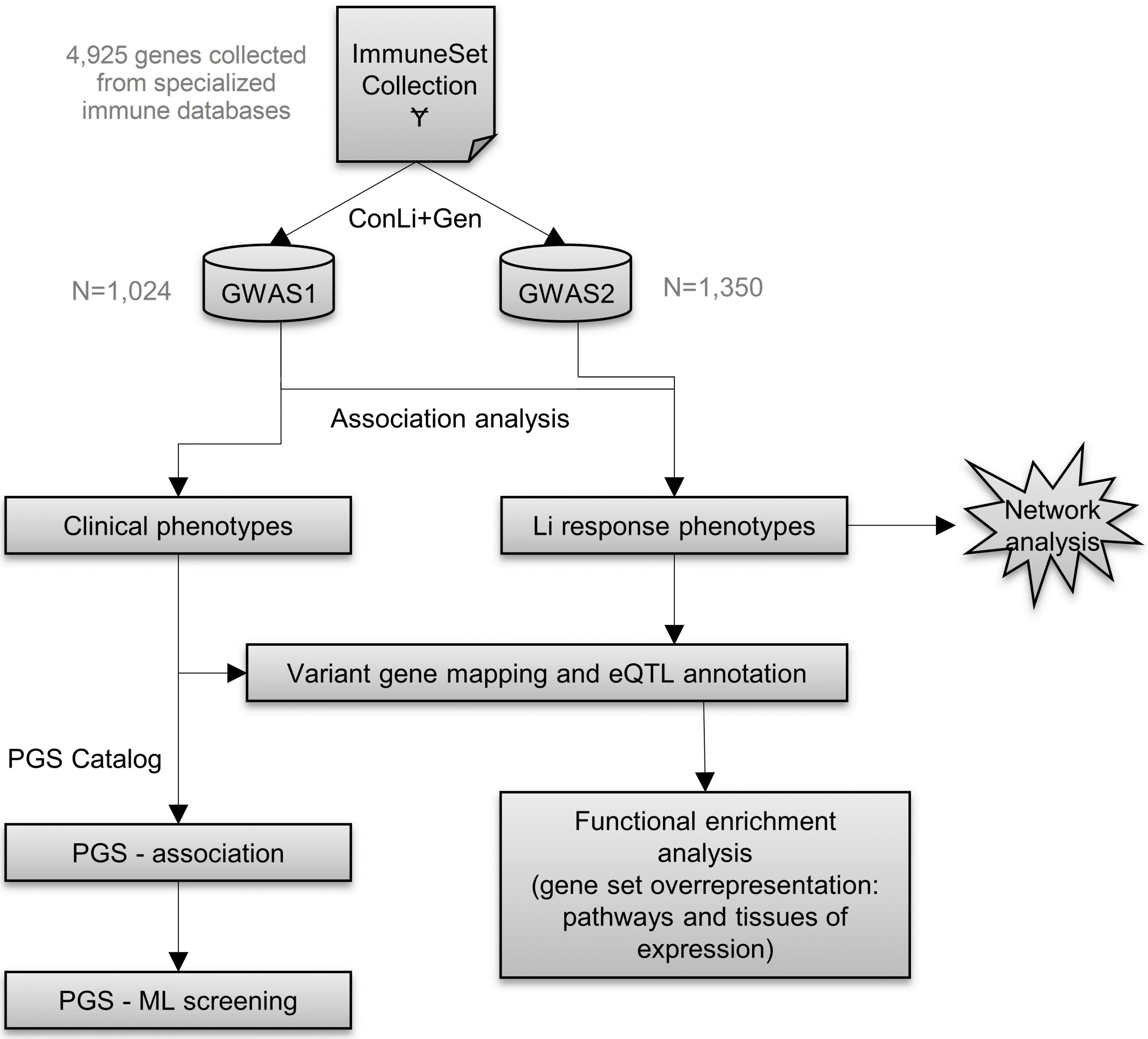

Figure S1. Workflow diagram. The association of 4,925 immune-related genes with lithium (Li) response phenotypes (i.e. Dichotomized response variable, continuous variable, Alda A, Alda B and total Alda) was investigated in ConLi$^+$Gen „GWAS1“ and „GWAS2“ samples. A network analysis for the dichotomized and continuous Li response phenotypes was performed to obtain biological insights. Variants were annotated for mapped genes and known expression quantitative trait loci (eQTL) effects in brain, blood, spleen and thyroid tissues, and immune cells. Mapped and mapped + eQTL gene lists underwent overrepresentation analyses of biological pathways and tissues of expression. In „GWAS1“, the association with clinical phenotypes in bipolar disorders (BP) was explored through association analysis as described for Li response. In addition, polygenic scores (PGS) for immune-related traits were obtained from PGS Catalog and calculated in ConLi$^+$Gen „GWAS1“ individuals to test for association with clinical phenotypes in BP (i.e. Dichotomized and continuous Li response variables, age-at-onset of disease, number of episodes of depression, hypomania and mania, and presence of psychosis, alcohol/substance use disorder and suicidal ideation). Finally, the calculated

PGSs were subjected to a supervised machine learning screening to obtain the relative importance of each PGS for Li response (dichotomized variable).

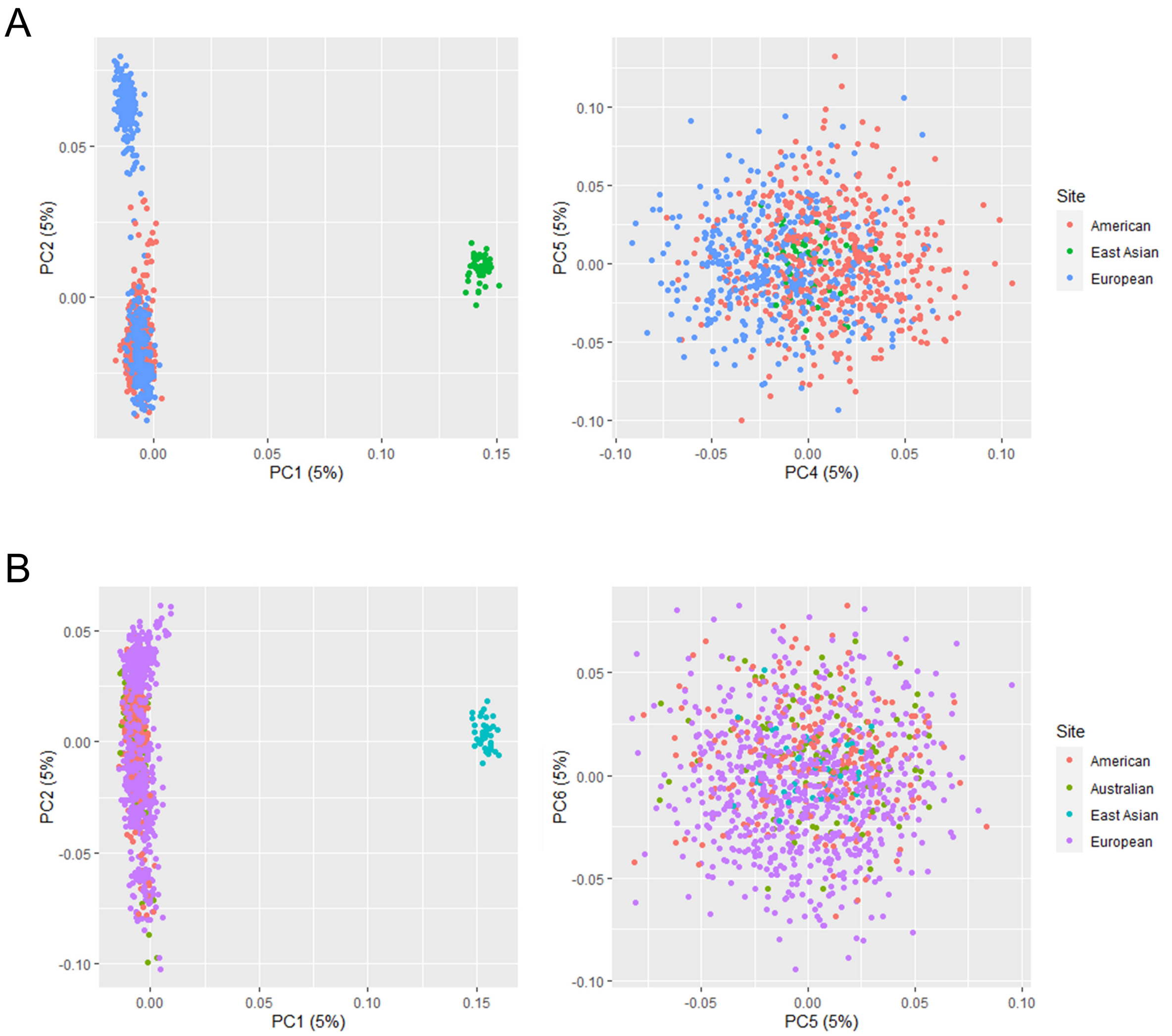

Figure S2. Principal component analysis. Plotted principal components (PCs) of the genetic data revealed that the first PC reflected ancestry (i.e. European or East Asian) in both ConLi+Gen samples. However, all stratification by this and other factors, such as recruitment site, is completely eliminated in both GWAS1 (A) and GWAS2 (B) ConLi+Gen samples in the first 5-6 PCs.

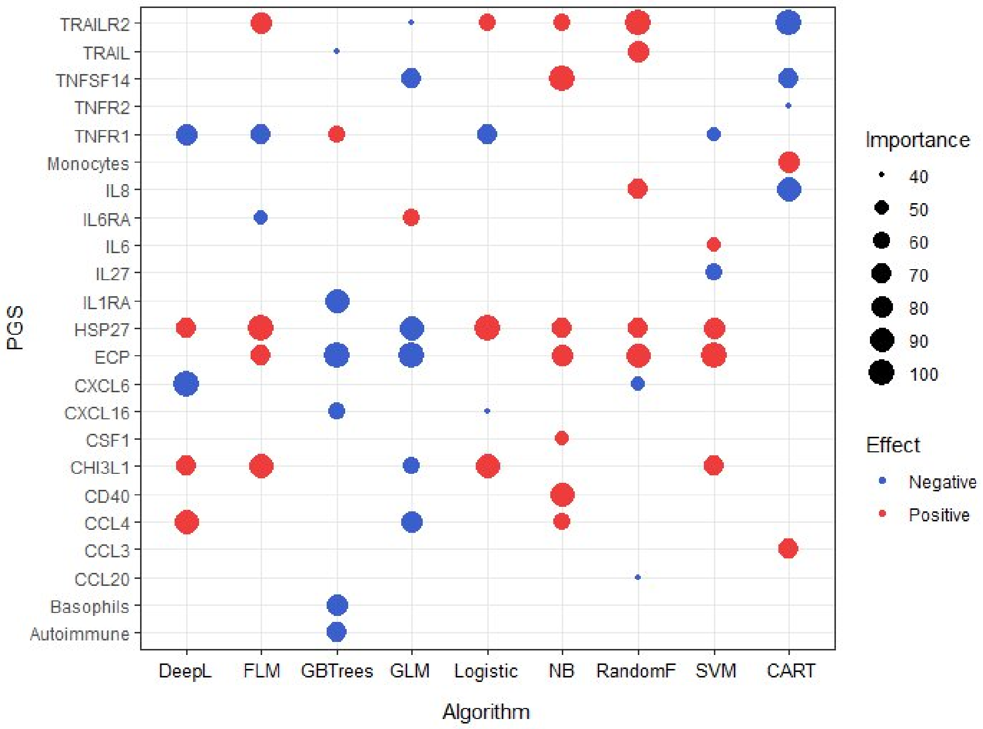

Figure S3. Machine learning PGS screening. Relative importance of calculated PGSs for the dichotomized response to Li treatment in ConLi+Gen "GWAS1". Various machine-learning algorithms were tested. The relative importance obtained with each algorithm is represented by the size of the bubbles. The direction of effect is colored in red when positive (i.e. favors response) and in blue when negative (i.e. favors non-response). DeepL: deep learning, FLM: fast large margin, GBTrees: gradient boosted trees, GLM: generalized linear model, Logistic: logistic regression, NB: naïve Bayes, RandomF: random forest, SVM: support vector machine, CART: decision tree.
